# Orphan Medicinal Products for the Treatment of Pancreatic Cancer: Lessons Learned From Two Decades of Orphan Designation

**DOI:** 10.3389/fonc.2021.809035

**Published:** 2021-12-20

**Authors:** Jorn Mulder, Tobias van Rossum, Segundo Mariz, Armando Magrelli, Anthonius de Boer, Anna M. G. Pasmooij, Violeta Stoyanova-Beninska

**Affiliations:** ^1^ Medicines Evaluation Board, Utrecht, Netherlands; ^2^ European Medicines Agency, Amsterdam, Netherlands; ^3^ National Center for Drug Research and Evaluation, Istituto Superiore di Sanità, Rome, Italy; ^4^ Division of Pharmacoepidemiology and Clinical Pharmacology, Utrecht Institute of Pharmaceutical Sciences, Utrecht, Netherlands

**Keywords:** orphan designation, rare disease, pancreatic cancer, european medicines agency, drug development, committee for orphan medicinal products

## Abstract

Pancreatic cancer has a dismal prognosis and only a few treatment options are available. In the European Union, pancreatic cancer classifies as a rare disease, allowing drug developers to apply for orphan medicinal product (OMP) designation. The aim of this study was to provide more detail on OMPs for pancreatic cancer. All applications for OMP designation submitted to the EMA between 2000 and 2019 were identified. For each medicinal product that received an OMP designation, the mode of drug action, use of protocol assistance, and current life cycle status was determined. Fifty-two medicinal products received an OMP designation. At the time of submission, eighteen OMPs were at the non-clinical and 34 OMPs were at the clinical stage of development. At least fourteen kinds of mode of action were explored in the condition. For eighteen out of 52 OMPs protocol assistance was sought. At the time of data analysis, one OMP received marketing authorisation and 24 OMPs were ongoing in development. Many medicinal products for pancreatic cancer received an OMP designation and the majority of these products was already in the clinical stage of development. Nonetheless, the success rate of OMPs for pancreatic cancer that reach the market is low, and increasing this rate is something to aspire. Fortunately, development is still ongoing for a part of the OMPs, and a few developers are planning to submit a marketing authorisation application in the near future. This however does not guarantee success, as pancreatic cancer remains a difficult disease to treat. Developers are advised to make optimal use of incentives such as protocol assistance, establishing (early) dialogue between regulators and drug developers and to agree on important topics such as clinical trial design.

## Introduction

Pancreatic cancer has a poor prognosis and is currently the seventh leading cause of cancer-related deaths worldwide (1). The most common type of pancreatic cancer is pancreatic ductal adenocarcinoma (2), and many patients are diagnosed when the cancer is already in the advanced stage of the disease (3). A reason for late diagnosis is that patients often do not experience any symptoms in the earlier stages of the disease (4, 5).

A few treatment options exist for patients with pancreatic cancer. Curative treatment is only optional in those that have a resectable tumour at the time of diagnosis; the minority of patients. Palliative treatment can be considered for patients with advanced or metastatic disease. Dependent on the performance status (PS) of the patient, FOLFIRINOX (PS 0 or 1), albumin-bound paclitaxel in combination with gemcitabine (PS 0 or 1) or gemcitabine monotherapy (PS 2 and/or bilirubin higher than 1.5 x upper limit normal) can be considered as a first-line treatment option, according to clinical practice guidelines (6). The only recommended second-line treatment option is liposomal irinotecan in combination with 5-fluoruracil (7). The median overall survival for first-line therapy varies between 6 and 11 months, dependent on the therapy that is administered (8). Despite available therapies, overall survival is generally poor, as reflected by the median OS being less than 1 year in patients with advanced pancreatic cancer. Hence, there is a clear unmet medical need.

According to the European Union Orphan Regulation, pancreatic cancer is classified as a rare disease (9), allowing drug developers to submit an application for orphan medicinal product (OMP) designation to the European Medicines Agency (EMA). Drug developers can submit an application for OMP designation if their product meets a couple of criteria. These criteria concern the seriousness of the disease, the prevalence of the disease, and the existence of a satisfactory method of diagnosis, prevention or treatment of the condition. Once an application is submitted to the EMA, the Committee for Orphan Medicinal Products (COMP) – one of the committees of the EMA – will examine the application. The final COMP opinion on OMP designation will be send to the European Commission (EC), and the EC decides whether the OMP designation will be granted (10). A range of incentives is offered by the EC through the Orphan Regulation. These incentives include protocol assistance (PA), fee reductions for regulatory procedures and market exclusivity (11). Protocol assistance is a kind of scientific advice specifically for OMPs (12). The aim of the Orphan Regulation is to stimulate research and development of medicinal products for rare diseases and ensure that effective medicinal products are authorised for diseases with a high unmet medical need.

To date, the COMP has approximately 20 years of experience with applications for OMP designation for pancreatic cancer. Through the years, many applications have been submitted to the EMA, and we are of the opinion that this orphan condition deserves further attention. The aim of this study was to provide a detailed overview on OMPs for pancreatic cancer, which can be of value for various stakeholders, including regulators and drug developers. Of special interest were the use of PA incentive and the current life cycle status.

## Methods

### Data Sources

Internal and publicly available documents from the EMA were used in this study. Internal data was derived from EMA/COMP summary reports on applications for OMP designation, PA letters, and annual reports on designated OMPs. Publicly available data was retrieved from public summaries of positive opinion for orphan designation and European public assessment reports (EPARs); both available at www.ema.europa.eu.

### Data Collection

All applications for OMP designation for medicinal products for the treatment of pancreatic cancer submitted to the COMP between 17 April 2000 and 31 December 2019 were included in this study.

From the summary reports the following information was obtained: date of submission, final COMP opinion, MoA, and stage of development at time of submission. In addition to the summary reports, information on MoAs was also obtained from public summaries. If the MoA was not clearly described in the summary report and/or public summary, literature describing the MoA was sought *via* PubMed.

PA letters were used to determine how many developers made use of this incentive and if advice on clinical development was sought.

From the annual reports the (development) status and the planned submission date was subtracted.

EPARs provided insight in the number of marketing authorisation applications (MAAs) submitted to the EMA. The time from OMP designation to Committee for Medicinal Products for Human Use (CHMP) opinion or withdrawal was determined by calculating the days between the date of the OMP designation and the date of final CHMP opinion or withdrawal of the MAA. Public summaries enabled the identification of OMPs that were withdrawn from the Community Register of designated Orphan Medicinal Products (access date: 12 March 2021).

### Statistics

Descriptive statistics were used.

## Results

### Applications for OMP Designation

Between 2000 and 2019, a total of 80 applications for OMP designation for pancreatic cancer were evaluated by the COMP. Of the 80 applications, 52 received a positive opinion on OMP designation, two received a negative opinion on OMP designation and 26 were withdrawn by the applicant prior to final COMP opinion. Seven applications were resubmitted to the agency after the first application was withdrawn; six applications were resubmitted once and one application was resubmitted twice. Of these, six were granted positive opinion on OMP designation; these positive opinions were already included in the total number of positive opinions mentioned above. The other application resulted in a second withdrawal and eventually a negative opinion; this negative opinion was already included in the total number of negative opinions mentioned above. All medicinal products that received positive opinion by the COMP were granted OMP designation by the EC (**Supplementary Table 1**).

### Simplified Mode of Action

**Table 1** shows the simplified MoAs of the OMPs for pancreatic cancer. The OMPs either ‘stimulate an immune response’; ‘block signalling pathway(s)’; ‘inhibit DNA synthesis’; ‘infiltrate tumour cells and replicate therein’; ‘improve the effectiveness of existing medicinal products’; ‘induce DNA lesions’; ‘counter migration of tumour cells’; ‘induce cell cycle arrest’; ‘deplete hyaluronan in tumour stroma’; ‘deplete an essential amino acid required for cell growth’; ‘deliver radiation specifically to tumour cells’; ‘collapse mitochondrial metabolism’; ‘trigger apoptosis’; or ‘induces oxidative stress’. The remaining OMPs had multiple MoAs. Additional information on the MoA can be found in **Supplementary Table 1**.

**Table 1:** Mode of drug action of OMPs for the treatment of pancreatic cancer.

Mode of drug action (simplified)	Number of OMPs
Stimulates an immune response	12
Blocks signalling pathway(s)	8
Inhibits DNA synthesis	5
Infects tumor cells and replicates therein	5
Improves the effectiveness of existing medicinal products	4
Multiple mechanisms	4
Induces DNA lesions	3
Counters migration of tumor cells	2
Induces cell cycle arrest	2
Delivers radiation specifically to tumour cells	2
Depletes hyaluronan in tumour stroma	1
Depletes an essential amino acid required for cell growth	1
Collapses mitochondrial metabolism	1
Triggers apoptosis	1
Induces oxidative stress	1

### Stage of Drug Development at Time of Orphan Designation

**Figure 1:**
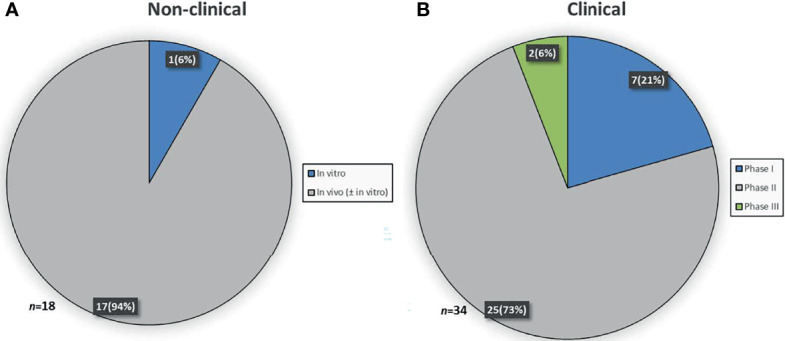
Stage of development at time of designation. **(A)** Study(ies) conducted in the non-clinical stage of development. **(B)** Latest study ongoing or completed in the clinical stage of development.

To determine which data was considered sufficient to grant OMP designation, the stage of development was identified for the 52 OMPs. At the time of submission, 18 medicinal products were at the non-clinical and 34 medicinal products were at the clinical stage of development. For the medicinal products in the non-clinical stage of development, one was investigated in an *in vitro* study and 17 were investigated in one or more *in vivo* ± *in vitro* studies (**Figure 1A**). For the medicinal products in the clinical stage of development, phase I, II and III clinical trials were ongoing/completed for 7, 25 and 2 medicinal products, respectively (**Figure 1B**).

### Use of Incentives

For 18 OMPs PA on the development of the product was sought. In total, PA was requested 23 times, including two follow-up advices and three additional advices for products for which PA was already requested previously. Nineteen of the PA requests contained questions concerning the clinical development of the OMP. Of these, twelve contained questions concerning a planned phase III trial. For four OMPs a question on a conditional marketing authorisation was included in the PA. For six OMPs a question on significant benefit was included in the PA.

### Current Status of the Orphan Medicinal Products

**Figure 2:**
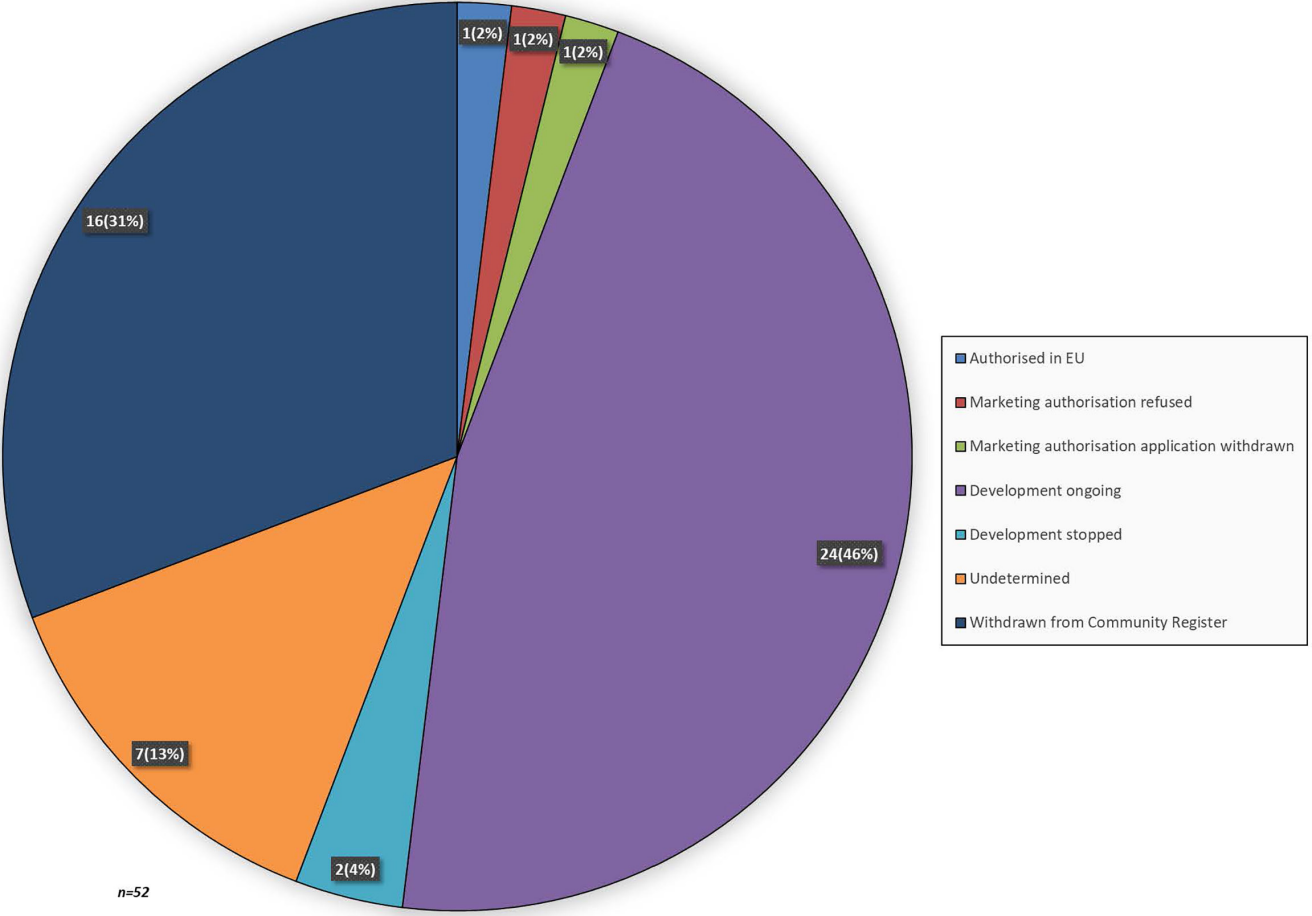
Lifecycle status of medicinal products that received an OMP designation for pancreatic cancer. When a recent annual report was absent the development lifecycle status was labelled as undetermined.

At the time of analysis, 36 medicinal products still had an OMP designation and 16 medicinal products were withdrawn from the EC Community Register. Of the medicinal products that still had an OMP designation, 1 was authorised in the EU for the treatment of pancreatic cancer, namely Onyvide (**Figure 2**). For two OMPs, Masiviera and Orathecin, a MAA was submitted to the EMA, but these applications did not result in a marketing authorisation. For Onyvide, Masiviera and Orathecin, the time from OMP designation to final CHMP opinion or withdrawal of the MAA was 1687, 1669, and 955 days, respectively. The development status was determined for the remaining 33 OMPs. Development was ongoing for 24 OMPs, stopped for 2 OMPs and could not be determined for 7 OMPs. Development was stopped due to financial or strategic reasons. Development status was undetermined due to the absence of an annual report, while still being included in the community register.

### Ongoing OMPS and Planned Submissions

**Figure 3:**
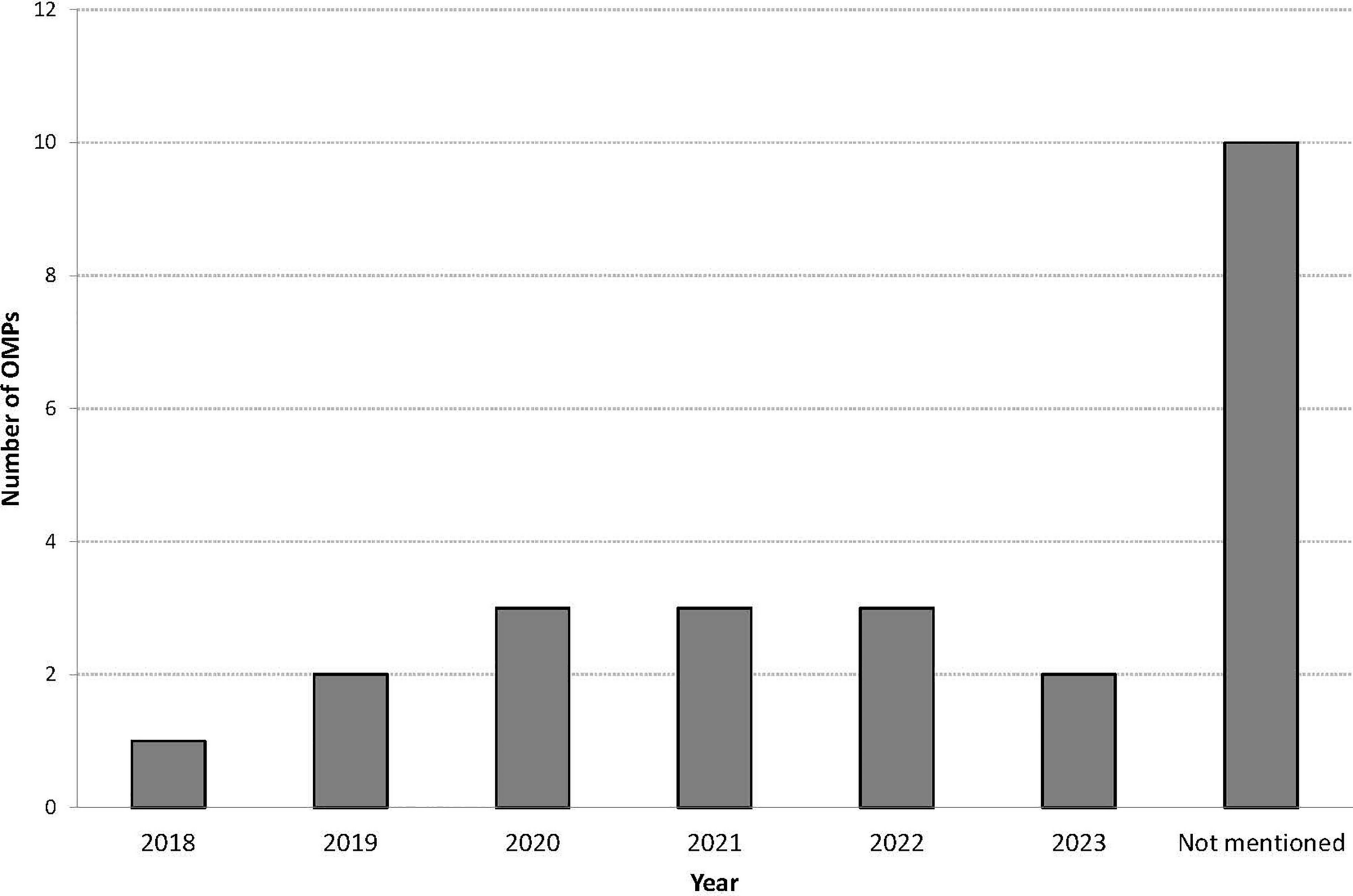
Planned submission date for an application for MA for OMPs ongoing in development.

A planned submission date for a MAA was included in the latest annual report for 14 out of 24 OMPs that were ongoing in development. Of the fourteen annual reports that included a planned submission date, six developers planned to submit a MAA before 2021 and eight developers planned to submit a MAA in 2021 or thereafter (**Figure 3**). The remaining sponsors did not specify a planned submission date.

## Discussion

To date, the COMP has two decades of experience with OMPs for pancreatic cancer, which prompted our interest in these products and their life cycle status. Through the years, a total of 52 medical products for pancreatic cancer were granted OMP designation. The major findings regarding these OMPs will be discussed in detail below.

Many of the medicinal products (65%) were already in the clinical stage of development when the developers applied for an OMP designation. This finding is however not solely confined to OMPs for the treatment of pancreatic cancer. Pauwels and colleagues revealed that the majority of anti-cancer medicinal products were in the clinical stage of development at the time of submission for OMP designation (13). Additionally, Mariz and colleagues showed that 68% of the applications for OMP designation were supported by preliminary clinical data (14). It may appear promising that many of the OMPs are already in the clinical stage of development, but it should be noted that the later stages of clinical development are often the most challenging. Hence, success cannot be guaranteed, in spite of encouraging non-clinical and preliminary clinical data. This is particularly the case for pancreatic cancer, as it is a notoriously difficult disease to treat with a high failure rate in drug development (15).

Our results show that OMPs for pancreatic cancer had distinct MoAs. The most commonly investigated OMPs included those that stimulate an immune response, block signalling pathways, infect tumour cells and replicate therein, and inhibit DNA synthesis. These OMPs can be classified as immunotherapy, targeted therapy, oncolytic virus therapy and chemotherapy, respectively. Chemotherapy continues to play an important role in the treatment of pancreatic cancer. However, other types of therapy have unfortunately not yet demonstrated definitive efficacy in pancreatic cancer, which concerns both OMPs as well as medicinal products without an OMP designation. Targeted therapy could be considered an exception, as a phase III clinical trial showed a statistically significant improvement in overall survival for erlotinib plus gemcitabine compared to gemcitabine monotherapy (16). However, the clinical relevance of this outcome is questioned, as the gain in median overall survival is approximately 2 weeks (6). There are several reasons why pancreatic cancer is such a difficult disease to treat. For instance, it is reported that a considerable part of the tumour mass is made up of a highly fibrotic stroma and this is associated with poor survival outcome (17). Furthermore, within the stroma, macrophages and inflammatory cells construct an immunosuppressive microenvironment, preventing an anti-tumour immune response (18, 19). Developing effective medicinal products remains challenging, despite the attempts to overcome these hurdles, as also seen by the MoAs of the OMPs included in this study. Therefore, a better understanding of the disease remains of importance.

To stimulate the development of medicinal products for rare diseases, incentives have been implemented in the EU Orphan Drug legislation (20). We found that PA, one of these incentives, was sought only for the minority of OMPs (35%). Moreover, almost all of the PAs requests included questions on the clinical development, including questions on the design of phase III clinical trials. Hence, it appears that developers are more likely to seek PA when their product is transitioning to the late stage of clinical development. This is not surprising, as agreement between regulators and developers on the design of phase III trials – the confirmatory trial – is of importance when considering potential future MAAs. There might be several reasons why not all of the developers have requested PA, including no advancement in development, financial limitations, or lack of efficacy in previously ongoing clinical trials. Besides, developers might not be aware of the benefit of PA and hence do not make use of this incentive. An analysis performed by Hofer and colleagues showed that compliance with PA was associated with a higher probability for MA. They advised that drug developers should make use of the incentive, as the development plan could be discussed and amended. This may prevent major outstanding issues during the evaluation of a MAA (21). Therefore, it remains important that developers continue to seek PA, considering the benefit of this incentive.

Even though the majority of medicinal products was already in the clinical stage of development when the developers applied for OMP designation, to date, only one OMP for pancreatic cancer received MA, namely irinotecan hydrochloride trihydrate (22). Irrespective of orphan condition, the success rate of medical products that reach the market as OMPs is estimated to be 8% (23), which is four times higher than our finding. These data highlight that – despite the efforts of developers – not many OMPs eventually will reach the market, especially not those for pancreatic cancer. Nonetheless, the lower success rate is of course related to the difficulties in treating the condition. This is further highlighted by the fact that the CHMP was of the opinion that the benefit-risk balance was not considered positive for two out of three OMP for pancreatic cancer considered for MA, namely rubitecan and masitinib (24, 25). This resulted in a withdrawal of MA application and a refusal on MA, respectively.

A positive finding in our results is that development is still ongoing for almost half of the OMPs (46%), and a couple of developers are even planning to submit an application for MA in the near future. Of all these, a few developers indicated their plans to submit a MAA in previous years, but this did not happen so far. The reasons for this might be delayed of failed development. For the remaining OMPs it could not be determined whether development is still ongoing, as the annual reports were absent or OMPs were withdrawn from the Community Register. It remains difficult to speculate on the reasons behind this, but plausible reasons could be either failure in development or financial considerations. At least for those products that have received an OMP designation a while ago.

This study has a few limitations, one of which is the lack of correction for time. For example, some medicinal products have received an OMP designation recently, while others have received OMP designation years ago. Therefore, products that have recently been granted OMP designation might still face potential developmental challenges in the future. Another limitation is the incompleteness of our overview on status of drug development, which is due to the lack of (recent) annual reports for a part of the OMPs. Determining whether the OMP is still in the drug pipeline of the developer would provide a more definitive answer on the life cycle status than is currently provided in our study.

## Conclusion

The success rate of medical products for pancreatic cancer that reach the market as OMPs is lower than for OMPs in general and increasing this success rate is something to aspire. Despite that pancreatic cancer is such a difficult disease to treat, a substantial number of applications has been submitted to the EMA for this condition, which indicates interest among drug developers. Development is still ongoing for a part of the OMPs, and for a few of these OMPs a submission for MAA in planned in the near future. It should be reminded that an OMP designation is supported by promising non-clinical and/or preliminary clinical data, but efficacy and safety still needs to be determined and the late stages of development are often the most challenging. Therefore an OMP designation is not a guarantee for a successful MA. In this respect, developers are advised to make optimal use of incentives inherent with an OMP designation, such as PA, establishing (early) dialogue between regulators and drug developers to agree on important topics such as clinical trial design. In addition, developers are strongly encouraged to provide yearly updates on advancements in development. Close monitoring of the drug development through the annual reports and transparency regarding the reason(s) for stopping development are crucial for saving human and financial resources and redirecting efforts in promising concepts.

## Data Availability Statement

The data analyzed in this study is subject to the following licenses/restrictions, the data that support the findings of this study are only available from the EMA through a confidentiality agreement which the authors signed. Requests to access these datasets should be directed to science@cbg-meb.nl.

## Author Contributions

JM, AP, and VS-B designed the study. JM and TV performed the research. JM, AP, and VS-B analysed the data. JM, TV, SM, AM, AD, AP, and VS-B wrote the manuscript. All authors contributed to the article and approved the submitted version.

## Author Disclaimer

The views expressed in this article are the personal views of the authors and may not be understood or quoted as being made on behalf of, or reflecting the position of the EMA or one of its committees, working parties, or any of the national agencies.

## Conflict of Interest

The authors declare that the research was conducted in the absence of any commercial or financial relationships that could be construed as a potential conflict of interest.

## Publisher’s Note

All claims expressed in this article are solely those of the authors and do not necessarily represent those of their affiliated organizations, or those of the publisher, the editors and the reviewers. Any product that may be evaluated in this article, or claim that may be made by its manufacturer, is not guaranteed or endorsed by the publisher.
