# Supplementary material for: Orphan Medicinal Products for the Treatment of Pancreatic Cancer: Lessons Learned From Two Decades of Orphan Designation
Source: Front Oncol. 2021 Dec 20;11:809035. doi: 10.3389/fonc.2021.809035 (PMC8720999; doi:10.3389/fonc.2021.809035)
Supplement: Supplementary file 1 [file Table_1.docx]

**Supplementary Table 1. Medicinal products for pancreatic cancer that were granted OMP designation between 2000 and 2019 (source: [www.ema.europa.eu](http://www.ema.europa.eu))**

| **Active substance** | **Date of approval** | **How is the medicine expected to work** | **Withdrawn** | **EMA hyperlink** |
| --- | --- | --- | --- | --- |
| Human reovirus type 3 Dearing strain | 24-04-2015 | This medicine is made up of an 'oncolytic' virus called reovirus that it is able to target, infect and destroy cancer cells but does not infect normal cells. When inside a cancer cell, the virus is expected to take over the cell's replication apparatus and use it to make more copies of itself. This is expected to kill the cell, leaving the virus to spread to neighbouring cancer cells. | No | <https://www.ema.europa.eu/en/medicines/human/orphan-designations/eu3151477> |
| Glufosfamide | 15-04-2011 | Glufosfamide is made of another anticancer medicine called isophosphoramide mustard linked to glucose (a sugar). It is a cytotoxic (cell-killing) substance that belongs to the group 'alkylating agents'. Alkylating agents kill cancer cells by attaching to their DNA while they are reproducing. As a result, cancer cells cannot reproduce and this slows down the growth of tumours. The glucose in the medicine is expected to help glufosfamide enter the tumour cells. | No | <https://www.ema.europa.eu/en/medicines/human/orphan-designations/eu311851> |
| Autologous human adipose perivascular stromal cells genetically modified to secrete soluble tumour necrosis factor-related apoptosis-inducing ligand | 19-11-2018 | The medicine is made of cells that have been modified to produce TRAIL, a protein in the body that triggers cancer cell death. When the medicine is injected into the patient’s cancer, the cells in the medicine will produce the TRAIL protein, which will attach to receptors (targets) on the surface of cancer cells and trigger their death. | No | <https://www.ema.europa.eu/en/medicines/human/orphan-designations/eu3182085> |
| Cysteamine bitartrate | 26-03-2014 | The active substance in this medicine, cysteamine bitartrate, is thought to block the action of certain enzymes called matrix metalloproteinases. Tumours such as pancreatic cancer produce high levels of these enzymes, which break down the substances that cement normal cells together in the tissues, enabling the cancer cells to spread more easily between them. By blocking the action of the enzymes, the medicine is expected to reduce the spread of cancer cells. | No | <https://www.ema.europa.eu/en/medicines/human/orphan-designations/eu3141252> |
| Heat-killed Mycobacterium obuense (whole cell) | 16-12-2014 | In the early stages of cancer, the body's immune system (its natural defences) can often combat the growth and spread of tumour cells, but over time the immune system may become less effective in controlling the cancer, allowing it to grow. This medicine contains a species of bacteria called Mycobacterium obuense (NCTC 13365) that have been killed by heating so they can no longer grow or cause infection. When the medicine is injected, the body's immune system is activated to become more effective, because the bacteria are considered a possible new threat. This activation of the immune system is expected to help the body also combat the cancer more effectively. | No | <https://www.ema.europa.eu/en/medicines/human/orphan-designations/eu3141385> |
| Immunoglobulin G1, anti-(human tumour-associated calcium signal transducer 2)(human-Mus musculus monoclonal hRS7 heavy chain), disulfide with human-Mus musculus monoclonal hRS7 k-chain, dimer, hexakis(thioether) with (4S)-4-[[[[4-[[(2S)-2-(4-aminobutyl)-2-[[2-[2-[[26-[4-[[[[4-[(3-mercapto-2,5-dioxo-1-pyrrolidinyl)methyl]cyclohexyl]carbonyl]amino]methyl]-1H-1,2,3-triazol-1-yl]-3,6,9,12,15,18,21,24-octaoxahexacos-1-yl]amino]-2-oxoethoxy]acetyl]amino]-1-oxoethyl]amino]phenyl]methoxy]carbonyl]oxy]-4,11-diethyl-9-hydroxy-1H-pyrano[3',4':6,7]indolizino[1,2-b]quinoline-3,14(4H,12H)-dione | 15-10-2014 | This medicine contains a monoclonal antibody (a type of protein) that is able to recognise and attach to a target substance called Trop-2, which is found on the surface of pancreatic cancer cells. The antibody has been linked to the activated form of an existing cancer medicine called irinotecan. When the antibody attaches to Trop-2, the activated irinotecan is delivered into the cancer cells where it blocks the action of topoisomerase I, an enzyme needed for the cells to divide and grow, and so leads to their death. By specifically targeting the cancer cells, side effects on normal cells should be reduced. | No | <https://www.ema.europa.eu/en/medicines/human/orphan-designations/eu3141343> |
| Herpes simplex type 1 virus containing cellular B-myb gene as tumour-specific promoter | 15-01-2015 | This medicine consists of an 'oncolytic' virus (a virus that kills cancer cells) that contains a part of a gene (called B-myb). This is expected to allow the virus to multiply and kill only cells that have high levels of the gene B-myb (such as pancreatic cancer cells), while sparing normal cells.  The virus in the medicine (a herpes virus) has been modified so that it does not cause disease in humans. | No | <https://www.ema.europa.eu/en/medicines/human/orphan-designations/eu3141412> |
| Live attenuated Listeria monocytogenes delta actA/delta inlB strain expressing human mesothelin | 11-01-2016 | The medicine works by stimulating the patient's immune system, the body's natural defences, so that it targets and destroys the cancer cells. It is made of Listeria monocytogenes bacteria, which have been attenuated (weakened) so that they do not cause disease in humans. The bacteria have also been modified to produce mesothelin. Mesothelin is found at high levels on many types of cancer cells, including pancreatic cancer cells.  When the medicine is injected into the body, the patient's immune system learns to treat mesothelin as 'foreign' and is expected to destroy pancreatic cancer cells carrying mesothelin on their surface.  The medicine is given as part of a combination treatment including another medicine (called 'two allogeneic irradiated pancreatic tumour cell lines' or GVAX) which is also expected to stimulate the immune system to recognise pancreatic cancer cells as foreign and so causes the body to attack the cancer. | Yes | <https://www.ema.europa.eu/en/medicines/human/orphan-designations/eu3151603> |
| Two allogenic irradiated pancreatic tumour cell lines | 11-01-2016 | The medicine works by stimulating the patient's immune system, the body's natural defences, so that it targets and destroys the cancer cells. It is made of two types of pancreatic cancer cells that have been modified to produce granulocyte-macrophage colony stimulating factor (GM-CSF) and have been treated with radiation to prevent them from growing. GM-CSF stimulates the immune system to recognise as 'foreign' certain proteins, such as mesothelin, which are found at high levels on the surface of pancreatic cancer cells. This is expected to stimulate the immune system to destroy pancreatic cancer cells.  The medicine is given as part of a combination treatment including another medicine (called 'live attenuated Listeria monocytogenes delta actA/delta inlB strain expressing human mesothelin' or CRS-207) which helps the immune system to recognise mesothelin as foreign and so stimulate the body to attack the cancer. | Yes | <https://www.ema.europa.eu/en/medicines/human/orphan-designations/eu3151604> |
| Mixture of seven synthetic fragments consisting of p21 RAS peptides | 05-08-2011 | The medicine contains seven synthetic peptides (short chains of amino acids) that are also found in a protein called 'p21 RAS'. The peptides contain mutations which are normally only expressed by certain cancer cells such as pancreatic cancer cells. The medicine is expected to act as a vaccine by 'teaching' the specialised cells of the body's immune system called T cells (a type of white blood cell) to recognise the p21 RAS proteins containing the mutations. It is expected that this will lead the T cells to attack and kill the pancreatic cancer cells containing these proteins. | No | <https://www.ema.europa.eu/en/medicines/human/orphan-designations/eu311885> |
| Mixture of two allogeneic human pancreatic cancer cell lines stably transduced with a retroviral vector encoding the murine alpha-(1,3)-galactosyltransferase gene | 10-10-2012 | The medicine is expected to work as a 'cancer vaccine', by activating the patient's immune system (the body's natural defences) so that it attacks and kills the cancer cells. The medicine is made of pancreatic cancer cells that have been modified to produce an enzyme called alpha-(1,3)-galactosyltransferase. This enzyme is responsible for the production of substances called alpha-Gal, which are known to trigger an immune response. When the patient is given the vaccine, the patient's immune system is expected to stimulate an immune response not only against the cancer cells in the vaccine which contain alpha-Gal, but also against the cancer cells in the patients even though they do not contain alpha-Gal. | Yes | <https://www.ema.europa.eu/en/medicines/human/orphan-designations/eu3121048> |
| Brivudine | 28-01-2010 | Brivudine is expected to work by blocking the activity of a protein called 'heat shock protein 27 (Hsp27), which is found in high amounts in pancreatic cancer cells. Hsp27 is known to play a key role in 'chemoresistance'. This means that it makes cancer cells not respond ('resistant') to chemotherapy, helping them to survive. When brivudine is given together with other anticancer medicines, it is expected to prevent the cancers cells from developing resistance to the medicines, helping to treat the disease. | No | <https://www.ema.europa.eu/en/medicines/human/orphan-designations/eu309703> |
| Nanoparticle albumin-bound paclitaxel | 26-11-2010 | Paclitaxel, the active substance in nanoparticle albumin-bound paclitaxel, belongs to the group of anticancer medicines known as the 'taxanes'. Paclitaxel blocks the ability of cancer cells to break down their internal 'skeleton' that allows them to divide and multiply. With their skeleton still in place, the cells cannot divide and they eventually die.  Paclitaxel has been available as an anticancer medicine since 1993 but is not authorised for pancreatic cancer. Conventional types of paclitaxel contain substances that dissolve the paclitaxel, but which can cause hypersensitivity (allergic) reactions. Nanoparticle albumin-bound paclitaxel does not contain these substances. Instead, the paclitaxel is attached to a human protein called albumin in tiny particles known as 'nanoparticles'. This makes it easy to prepare a suspension of paclitaxel, which can be infused into a vein. The nanoparticles may also modify the way the medicine is distributed within the body, and this is expected to have a positive effect on its benefits and risks, in comparison with conventional medicines containing paclitaxel | Yes | <https://www.ema.europa.eu/en/medicines/human/orphan-designations/eu310809> |
| Polyinosine-polycytidylic acid coupled with the polycationic polyethyleneimine | 06-06-2012 | The medicine is expected to act against tumour cells by activating an enzyme within the cell called helicase MDA-5, which will cause the cell to digest and kill itself. Moreover, the medicine is expected to stimulate the immune system to recognise and attack tumour cells | Yes | <https://www.ema.europa.eu/en/medicines/human/orphan-designations/eu3121000> |
| (1-methyl-2-nitro-1H-imidazole-5-yl)methyl N,N’-bis(2-bromoethyl)diamidophosphate | 17-07-2013 | Pancreatic cancer, like most solid tumours, has areas with poor blood supply and therefore low levels of oxygen. The low level of oxygen in these areas is known to make tumour cells more resistant to standard chemotherapy. This medicine is expected to be converted into an active, toxic form called bromo-isophosphoramide mustard under conditions of low oxygen, allowing it to attack the tumour cells in low oxygen areas. This medicine is intended to be given with standard chemotherapy medicines and this is expected to help kill the tumour cells in low oxygen areas as well as other areas of the tumours. | Yes | <https://www.ema.europa.eu/en/medicines/human/orphan-designations/eu3131152> |
| 5'-O-(trans-9''-octadecenoyl)-1-beta-D-2'-deoxy-2',2'-difluorocytidine | 28-10-2009 | 5'-O-(trans-9''-octadecenoyl)-1-beta-D-2'-deoxy-2',2'-difluorocytidine belongs to the group ‘anti-metabolites’. In the body, this medicine is expected to be incorporated into the genetic material of cells (DNA and RNA) and interfere with the enzymes involved in making new DNA and RNA. As a result, it is expected to inhibit the growth of tumour cells and eventually kill them. | Yes | <https://www.ema.europa.eu/en/medicines/human/orphan-designations/eu309680> |
| 4-imino-1, 3-diazobicyclo-[3.1.0]-hexan-2-one | 27-07-2005 | Cells contain several substances needed for their normal functioning (e.g. glutathione, cysteine, thiols). They also contain small structures (so-called mitochondria), responsible for the production of the energy necessary for the cell functioning, through a process named “cellular respiration”. 4-imino-1, 3-diazobicyclo-[3.1.0] hexan-2-one might induce a certain reaction in the cancer cells leading to a shortage of the cell fundamental substances and the destruction of the surface of the mitochondria. This would then induce the destruction of the cell itself, through a process called “programmed cell death” (apoptosis). | No | <https://www.ema.europa.eu/en/medicines/human/orphan-designations/eu305299> |
| 4-amino-1-[(1S,4R,5S)-2-fluoro-4,5-dihydroxy-3-(hydroxymethyl)cyclopent-2-en-1-yl]pyrimidin-2-one | 12-10-2017 | The medicine is activated inside cancer cells by an enzyme called UCK2. Because this enzyme is present mainly in cancer cells, the medicine is activated only in these cells. The activated form of the medicine blocks the production of the cell's genetic material, RNA and DNA, and so kills the cancer cells. This is expected to slow down the growth of the cancer. | No | <https://www.ema.europa.eu/en/medicines/human/orphan-designations/eu3171937> |
| Chimeric monoclonal antibody against claudin-18 splice variant 2 | 05-08-2013 | Claudin-18 splice variant 2 is a protein found in the cells of the ducts of the pancreas, where it helps the cells to stick to each other. In patients with pancreatic cancer, this protein is produced in large amounts and is thought to be involved in the survival and spread of the cancer cells.  The medicine is a monoclonal antibody (a type of protein) that has been designed to recognise and attach to a part of the claudin-18 splice variant 2 protein in the cancer cells. By attaching to this protein, this medicine is expected to stimulate the immune system (the body's natural defences) to kill the cancer cells, slowing down the spread of the cancer. | No | <https://www.ema.europa.eu/en/medicines/human/orphan-designations/eu3131177> |
| L-asparaginase encapsulated in erythrocytes | 15-05-2009 | L-asparaginase is an enzyme that breaks down the substance L-asparagine, which is required for cell growth. Certain cancer cells, such as the cancerous cells in pancreatic cancer, cannot make L-asparagine, so they need to take it up from the blood in order to grow. By reducing the levels of L-asparagine in the blood, this medicine is expected to deprive the cancerous pancreatic cells of their supply of L-asparagine, causing them to die. L-asparaginase has already been used for the treatment of acute lymphoblastic leukaemia (a cancer of the white blood cells) since the 1970s. This medicine is made up of erythrocytes (red blood cells) that have been loaded with L-asparaginase so that the L-asparaginase is 'encapsulated' (contained) within the erythrocytes. The erythrocytes reduce the exposure of L-asparaginase to the immune system (the body's natural defences). This results in the immune system producing fewer antibodies against L-asparaginase, which could otherwise cause side effects such as allergic reactions. The erythrocytes also form tiny compartments where the breakdown of L-asparagine can take place. Together, these properties are expected to increase how long L-asparaginase remains active in the body and to allow a lower dose of the enzyme to be used for the same anticancer effect as the free enzyme. | No | <https://www.ema.europa.eu/en/medicines/human/orphan-designations/eu309633> |
| [5-amino-1-(4-fluoro-phenyl)-1H-pyrazol-4-yl]-[3-(2,3-dihydroxy-propoxy)-phenyl]-methanone | 22-08-2014 | This medicine is expected to work in patients with pancreatic cancer by blocking the action of proteins called p38 MAP kinases. In pancreatic cancer, p38 MAP kinases play an important role in regulating the way that cells of the immune system (the body's natural defences) respond to various chemical messengers from the cancer. By blocking p38 MAP kinases, the medicine is expected to improve the ability of the immune system to recognise and destroy cancer cells, thereby slowing the progression of the disease. | No | <https://www.ema.europa.eu/en/medicines/human/orphan-designations/eu3141323> |
| 6,8-bis(benzylthio)octanoic acid | 14-12-2018 | The medicine, which is taken up in large amounts by cancer cells, blocks two enzymes that are needed for mitochondria (the energy-producing components within cells) to work properly. As a result, the cells cannot produce the energy needed to survive and grow. This is expected to lead to the death of cancer cells in patients with pancreatic cancer and thereby slow down the growth of the cancer. | No | <https://www.ema.europa.eu/en/medicines/human/orphan-designations/eu3182105> |
| Adenoviral vector of serotype 5 modified to contain a chimeric sequence consisting of a minimal urokinase-type plasminogen activator receptor promoter preceded by three Notch-responsive elements, and coated with oligopeptide end-modified poly (beta-amino) esters | 16-10-2017 | This medicine is an advanced therapy that belongs to the group called 'gene therapy products'. These are medicines that work by delivering genes into the body.  The medicine is made up of an 'oncolytic' virus, a virus that has been genetically modified so that it is able to target, multiply in and destroy cancer cells while sparing normal cells. The virus is coated with a substance that protects it from the immune system (the body's natural defences) and allows it to be drawn to the cancer. When inside a cancer cell, the virus is expected to take over the cell's replication apparatus and use it to make more copies of itself. This is expected to kill the cell, leaving the virus to spread to neighbouring cancer cells. | No | <https://www.ema.europa.eu/en/medicines/human/orphan-designations/eu3171917> |
| Antroquinonol | 12-01-2017 | Antroquinonol is expected to work by blocking 'the Ras signalling pathway'. This is a mechanism within cells that helps them to grow and survive. However, in cancer cells it works abnormally, leading to the growth of the cancer. By blocking the Ras pathway, antroquinonol is expected to kill cancer cells and slow down the growth of the cancer. | No | <https://www.ema.europa.eu/en/medicines/human/orphan-designations/eu3161812> |
| Genetically modified human adenovirus encoding human PH20 hyaluronidase | 21-06-2011 | This medicine is made up of an 'oncolytic' virus, a virus that has been genetically modified so that it is able to target, replicate itself in and destroy tumour cells while sparing normal cells. When inside a tumour cell, the virus is expected to take over the cell's replication apparatus and use it to make more copies of itself. This is expected to kill the cell, leaving the virus to spread to neighbouring tumour cells. | No | <https://www.ema.europa.eu/en/medicines/human/orphan-designations/eu311880> |
| Salirasib | 21-06-2011 | Salirasib is expected to work by blocking 'the Ras signalling pathway'. This is a mechanism within cells that helps them to grow and survive. However, in cancer cells it works abnormally, leading to the growth of the cancer. Salirasib is expected to attach to specific proteins on the membrane of cancer cells and thus detach proteins called Ras. By detaching Ras, the medicine is expected to block the signalling pathway, thereby slowing down the growth of the cancer. | Yes | <https://www.ema.europa.eu/en/medicines/human/orphan-designations/eu311871> |
| Masitinib mesilate | 28-10-2009 | Masitinib mesylate is expected to work by blocking types of enzymes known as tyrosine kinases. These enzymes can be found in some receptors on the surface of cancer cells, including 'c-Kit' receptors and 'platelet-derived growth factor' (PDGF) receptors. These are receptors involved in stimulating the cells to divide uncontrollably. By blocking these receptors, masitinib mesylate is expected to help to control cell division, slowing down the rate of growth of the cancer. | No | <https://www.ema.europa.eu/en/medicines/human/orphan-designations/eu309684> |
| Nanoliposomal irinotecan | 09-12-2011 | Irinotecan is an anticancer medicine that belongs to the group 'topoisomerase inhibitors'. It blocks an enzyme called topoisomerase I, which is involved in the division of cell DNA. When the enzyme is blocked, the DNA strands break. This prevents the cancer cells from dividing and they eventually die.  Free irinotecan is already authorised for the treatment of colorectal cancer. In this medicine, irinotecan is contained within tiny fat particles called 'nanoliposomes'. The nanoliposomes are expected to accumulate within the tumour and release the medicine slowly over time, thereby decreasing the rate at which the irinotecan is removed from the body and allowing it to act for longer. | No | <https://www.ema.europa.eu/en/medicines/human/orphan-designations/eu311933> |
| N-[(2S)-2,3-dihydroxypropyl]-3-[(2-fluoro-4-iodophenyl)amino]isonicotinamide hydrochloride | 09-11-2009 | N-[(2S)-2,3-dihydroxypropyl]-3-[(2-fluoro-4-iodophenyl)amino]isonicotinamide hydrochloride is expected to work by blocking an enzyme called MEK1/2, which is involved in stimulating cells to grow and divide. MEK1/2 is over-activated in cancer cells, which makes these cells divide uncontrollably. By blocking this enzyme, the medicine is expected to control cell division and slow down the rate of growth of the cancer. | Yes | <https://www.ema.europa.eu/en/medicines/human/orphan-designations/eu309685> |
| Humanised IgG4 monoclonal antibody to the human toll-like receptor type 2 | 27-02-2017 | The medicine is a monoclonal antibody (a type of protein), that has been designed to attach to another protein called the human toll-like receptor type 2 (TLR2). TLR2 is part of the immune system (the body's natural defences) and may play a role in the spread of cancer. By attaching to TLR2, this medicine is expected to block its activity, thereby reducing cancer spread. | No | <https://www.ema.europa.eu/en/medicines/human/orphan-designations/eu3171838> |
| S-[2,3-bispalmitoyloxy-(2R)-propyl]-cysteinyl-GNNDESNISFKEK | 15-05-2009 | S-[2,3-bispalmitoyloxy-(2R)-propyl]-cysteinyl-GNNDESNISFKEK is a peptide (a protein fragment) that is expected to work by attaching to and activating two receptors called 'Toll-like receptor 2' and 'Toll-like receptor 6'. These receptors are part of the immune system (the body's natural defences) and their activation leads to an immune response. The medicine is injected directly into the pancreas, from where it is expected to stimulate the release of substances of the immune system that attack the cancer cells, particularly when it is combined with other cytotoxic (cell-killing) medicines. | No | <https://www.ema.europa.eu/en/medicines/human/orphan-designations/eu309634> |
| Recombinant human monoclonal antibody of the IgG1 kappa class against prostate stem cell antigen | 24-01-2013 | The medicine 'recombinant human monoclonal antibody of the IgG1 kappa class' is a monoclonal antibody, a type of protein that has been designed to recognise and attach to a specific structure (called an antigen) that is found in the body. It is expected to attach to an antigen called 'prostate stem=cell antigen', which is found on the surfaces of normal cells as well as in high amounts on the surface of prostate, bladder and pancreatic cancer cells. The exact function of prostate stem-cell antigen is not fully understood, although it is thought to play a role in regulating cell growth through regulating the transmission of chemical signals between cells. When the medicine attaches to prostate stem-cell antigen, it is expected to alter the antigen's normal activity, including blocking the transmission of chemical signals, thereby reducing the growth and spread of the pancreatic cancer cells. | Yes | <https://www.ema.europa.eu/en/medicines/human/orphan-designations/eu3121090> |
| Modified adenovirus serotype 5/35 containing a CMV promoter-driven transgene cassette with the human transgenes for a membrane-bound CD40 ligand and full length 4-1BBL | 28-07-2015 | The medicine is made up of an 'oncolytic' virus, a virus that has been modified so that it can target, infect and destroy cancer cells, but not normal cells. When inside a cancer cell, the virus is expected to take over the cell's replication apparatus and use it to make more copies of itself. This is expected to kill the cell, leaving the virus to spread to neighbouring cancer cells. In addition, the modifications to the virus prepare the cell for self-destruction and cause infected cells to produce substances that stimulate the immune system (the body's natural defences), which will also help to destroy the cancer cells. | No | <https://www.ema.europa.eu/en/medicines/human/orphan-designations/eu3151516> |
| Pegylated recombinant human interleukin-10 | 12-12-2016 | This medicine is a type of immunotherapy, which means that it acts on the body's immune system (the body's natural defences). It activates white blood cells known as CD8+ T cells. These cells can infiltrate pancreatic cancer tumours, where they are expected to kill cancer cells and improve survival. | No | <https://www.ema.europa.eu/en/medicines/human/orphan-designations/eu3161804> |
| Trabedersen | 24-07-2009 | Trabedersen is an 'antisense oligonucleotide', a short piece of DNA that has been designed to attach to the genetic material of cells responsible for producing a protein called TGF-β2. This blocks the production of TGF-β2. TGF-β2 is produced in large quantities in pancreatic cancer cells, and is involved in the growth, progression, and spreading of the cancer, as well as in the suppression of the body's immune system (the body's natural defences). By blocking the production of TGF-β2, trabedersen is expected to stop the cancer cells from growing and multiplying. In addition, blocking the production of TGF-β2 may stimulate the immune system to attack the cancer cells. | No | <https://www.ema.europa.eu/en/medicines/human/orphan-designations/eu309660> |
| Sodium 2-hydroxylinoleate | 23-08-2017 | This medicine is expected to work by reducing the activity of several proteins in the 'Akt/mTOR signalling pathway'. This is a mechanism within cells which is important in regulating their growth and survival. In many cancers, including pancreatic cancer, this pathway is overactive, allowing the cancer cells to grow uncontrollably. By reducing the activity of this pathway, the medicine is expected to slow down the progression of the cancer. | No | <https://www.ema.europa.eu/en/medicines/human/orphan-designations/eu3171911> |
| Pegylated recombinant human hyaluronidase PH20 | 16-12-2014 | The medicine contains a version of a natural enzyme, hyaluronidase, which breaks down a compound, hyaluronan. Hyaluronan is found in large amounts in many pancreatic cancers and helps the cancer to grow and to resist the effects of cancer medicines. By breaking down the excess hyaluronan, the medicine is expected to make the cancer easier to treat with other authorised therapies.  In this medicine, hyaluronidase has been 'pegylated' (combined with a chemical called polyethylene glycol). This decreases the rate at which the substance is removed from the body and allows the medicine to be given less often. | No | <https://www.ema.europa.eu/en/medicines/human/orphan-designations/eu3141394> |
| Anti-CEA sheep-human chimeric monoclonal antibody labeled with iodine-131 | 07-05-2003 | Antibodies are proteins that are able to distinguish certain foreign substances called antigens.  Examples of antigens are proteins found on the surface of cancer cells or bacteria. Anti-CEA sheephuman chimeric monoclonal antibody targets a substance present on the surface of pancreatic cancer cells. This substance is called carcinoembryonic antigen, or CEA. The antibody is called chimeric because it is composed of parts that were first found in different species of living things. In this case, the species are sheep, and man. It is called monoclonal because it is produced using cells that have identical genes, and produce exactly the same antibody. The antibody is also linked (labelled) with a tiny part, called iodine-131. Iodine-131 can give off radiation. Radiation can damage and kill cells, especially those that are dividing, such as cancer cells. Thus, the antibody is used to deliver the radiation to the pancreatic cancer cells and to kill them with the radiation. | Yes | <https://www.ema.europa.eu/en/medicines/human/orphan-designations/eu303142> |
| G17(9) gastrin-diphtheria toxoid conjugate | 24-01-2003 | Gastrin is a hormone that is normally produced by the body. Gastrin stimulates the stomach to produce gastric juice. Gastrin also makes certain digestive system cancers grow faster. G17(9) gastrin-diphtheria toxoid conjugate is designed to stimulate the body to make antibodies against gastrin. Antibodies are proteins which specifically recognise and block certain substances. Antibodies against gastrin are expected to specifically link to this hormone, thus blocking its activity. This would consequently slow down the growth of pancreatic cancer. | No | <https://www.ema.europa.eu/en/medicines/human/orphan-designations/eu302129> |
| Deuterium oxide | 20-10-2004 | Deuterium oxide is, in very small amounts, present in normal drinking water. In high amounts deuterium oxide might influence and block several processes in cancer cells necessary for tumour growth. Therefore it might prevent cancer cells from growing when given in higher concentrations. | Yes | <https://www.ema.europa.eu/en/medicines/human/orphan-designations/eu304239> |
| Cytochrome P450 isoform 2B1 gene transfected human embryonic kidney 293 cells encapsulated in polymeric cellulose sulphate | 30-06-2003 | This orphan medicinal product is made of cells whose genetic material has been modified by adding a gene. This gene allows the cells to be very efficient in activating an anti-cancer agent called ifosfamide. The active ifosfamide is then able to kill tumour cells. After having been genetically modified, the cells are packed into capsules, and the product is administered in a vessel near to the cancer. Polymeric cellulose sulphate is a material that is used so that the capsules get trapped into the small vessels of the tumour. Once the cells are in place, the agent ifosfamide is given. In this way, it is expected that the cells will be able to produce high levels of active ifosfamide near the tumour where it is most needed for killing the cancer cells. | No | <https://www.ema.europa.eu/en/medicines/human/orphan-designations/eu303149> |
| 5-10-Methylene-tetrahydrofolate | 11-06-2003 | 5,10-methylene-terahydrofolate belongs to a group of substances that are called folates. Folates, are necessary for the human body. They are obtained from the diet or from bacteria that live normally in the gut. They help in the building of new substances. Cancer cells need to build new genetic material in order to grow. Several proteins work in the cells in order to build the new genetic material. One of these proteins is called thymidylate synthase. It builds new genetic material using 5,10-methylene-terahydrofolate, which is transformed in the process. If fluorouracil is present, however, the transformation stops, and the protein is blocked. This damages the cells that are growing. Florouracil works best only if folates are present. As the folates from the diet may not be sufficient, it is expected that by giving additional 5,10-methylene-terahydrofolate, this will help fluorouracil to block the thymidylate synthase. Thus, giving 5,10-methylene-terahydrofolate together with fluorouracil could help to stop the growth of pancreatic cancer cells. | Yes | <https://www.ema.europa.eu/en/medicines/human/orphan-designations/eu303143> |
| 9-nitro-20 (S) camptothecin (Rubitecan) | 10-06-2003 | Rubitecan, belongs to a group of alkaloids called camptothecins. Alkaloids are substances that are naturally found in plants. Certain camptothecins are useful in medicine as anti-cancer agents. When cells are growing, as is the case for cancer cells, the genetic material (DNA) inside the cell may become twisted. Cells have several proteins which help to remove any twists in the DNA. This avoids that the DNA breaks, which would damage the cells. Camptothecins are able to block one of the proteins that can remove twists in the DNA. This protein is called topoisomerase I. By blocking this protein, rubitecan is expected to damage the cancer cells. | No | <https://www.ema.europa.eu/en/medicines/human/orphan-designations/eu303145> |
| 5,10-methylene-tetrahydrofolic acid | 02-09-2004 | 5,10-methylene-tetrahydrofolate belongs to a group of substances that are called folates. Folates, are necessary for the human body. They are obtained from the diet or from bacteria that live normally in the gut. They help in the building of new body substances. Cancer cells need to build new genetic material in order to grow. Several proteins work in the cells in order to build the new genetic material. One of these proteins is called thymidylate synthase. It builds new genetic material using 5,10-methylene-tetrahydrofolate, which is transformed in the process. If fluorouracil is present, however, the transformation stops, and the protein is blocked. This damages the cells that are growing, because the building of new genetic material becomes impossible. Fluorouracil works best only if folates are present. As the folates from the diet may not be sufficient, it is expected that by giving additional 5,10-methylene-tetrahydrofolate, this will help fluorouracil to block the thymidylate synthase. Thus, giving 5,10-methylene-tetrahydrofolate together with fluorouracil could help to stop the growth of pancreatic cancer cells. | Yes | <https://www.ema.europa.eu/en/medicines/human/orphan-designations/eu304221> |
| Bovine bile extract | 20-06-2005 | This medicinal product is a biological preparation isolated from the bile of cattle. The mechanism of action of bovine bile extract is not fully known. It is believed to activate a certain type of white blood cells, a type of cell belonging to the body's defence system (immune system), the so-called macrophages. The main role of macrophages is to take-up material (such as bacteria, cancer cells or cell fragments). Following the uptake they degrade the material and present parts of this material to the other components of the body's defence system. Activation of these cells may thus stimulate an immune response of the organism, which might cause the immune system to recognise and kill the cancer cells. | No | <https://www.ema.europa.eu/en/medicines/human/orphan-designations/eu305287> |
| Chimeric antibody to mesothelin | 17-03-2008 | Mesothelin is a protein that can be found on the surface of some normal cells and it is also found on the surface of cancer cells. Mesothelin and other substances that are present outside the cells interact with other substances in the cell surroundings and are part of the mechanisms that allow cells to grow and move (migrate). This is important for the development of cancers as these mechanisms are necessary for cancer growth and spreading. Antibodies are proteins in the body that target and bind specific structures on the surface of foreign bodies, such as bacteria or cancer cells. This antibody to mesothelin is able to bind mesothelin and interfere with its function on cell growth and migration. The product is expected to be able to inhibit cancer cell growth and spreading in the body. | Yes | <https://www.ema.europa.eu/en/medicines/human/orphan-designations/eu308536> |
| Nimotuzumab | 03-06-2008 | Nimotuzumab is an anticancer medicine that belongs to the group 'epidermal growth factor receptor (EGFR) inhibitors'. It blocks the receptors for a protein called 'epidermal growth factor', which are found on the surface of certain tumour cells. Epidermal growth factor normally stimulates cells to grow and divide. By blocking its receptor, nimotuzumab prevents the tumour cells receiving the messages they need for growth, progression and spreading. | No | <https://www.ema.europa.eu/en/medicines/human/orphan-designations/eu308550> |
| Paclitaxel (liposomal) | 31-10-2006 | When cells divide and grow, there are structures (tubules) inside the cells that need to assemble and disassemble in a very orderly way. Paclitaxel interferes with the assembly of these tubules and subsequently with the growth of cells. Liposomal paclitaxel is delivered to the cancer cells in the tumour in little lipid particles (liposomes) that bind specifically to the cells that line blood vessels. According to the sponsor, paclitaxel (liposomal) will, by inhibiting the growth of newly formed blood vessels, contribute to the destruction of the tumour. | No | <https://www.ema.europa.eu/en/medicines/human/orphan-designations/eu306419> |
| Yttrium (90Y)-DOTA-radiolabelled humanized monoclonal antibody against mucin 1 | 06-02-2009 | Mucin 1 is a protein found on the membrane of many types of cells. However, abnormally high levels or changes in its structure have been associated with some types of cancer and mucin 1 is highly expressed in pancreatic cancer cells. This medicinal product contains an antibody that specifically binds to mucin 1 and is labelled with radioisotope Yttrium (90Y)-DOTA.  Antibodies are proteins used by the immune system to identify and neutralize foreign proteins expressed by bacteria or viruses known as antigens. Antigens and antibodies have a lock-and-key relationship; an antibody can specifically recognize and bind only one antigen. As medicinal products, antibodies can be used to identify specific antigens expressed only on cells of interest. This medicinal product is expected to recognise mucin 1 on pancreatic cancer cells, bind on them and deliver the radioisotope on the tumour. Radioisotopes are used to destroy cancer cells. The antibody is expected to help identify pancreatic cancer cells expressing mucin-1, and once the product is bound, Yttrium (90Y)-DOTA is expected to destroy them. | Yes | <https://www.ema.europa.eu/en/medicines/human/orphan-designations/eu308608> |
| Human telomerase reverse transcriptase peptide (611-626) | 25-07-2006 | Human telomerase reverse transcriptase peptide (611-626) is a part of the enzyme (a protein that triggers chemical reactions) telomerase reverse transcriptase, which is often present in pancreatic cancer tumour cells. Human telomerase reverse transcriptase is needed for tumour cells to be able to divide many times (proliferate) and subsequently for the tumour to grow. The medicinal product is designed to activate the body's natural defence system, the immune system against the cells containing the human telomerase reverse transcriptase. According to the sponsor the product will trigger the immune system against the pancreatic cancer tumour cells, thus destroying the tumour cells with the body's own defence system. | No | <https://www.ema.europa.eu/en/medicines/human/orphan-designations/eu306384> |
| Cisplatin (liposomal) | 08-06-2007 | Cisplatin (liposomal) is a new formulation of cisplatin, which has been an anti-cancer drug for a long time. The inclusion of cisplatin in liposomes (special particles coated with molecules of fat) is expected to increase the concentration of the drug in the cancer cells, compared to normal cells, and decrease the adverse effects of the drug. Cisplatin works by damaging and eventually killing cancer cells, through the formation of special compounds, called “reactive oxygen species”, which are toxic for the cells | No | <https://www.ema.europa.eu/en/medicines/human/orphan-designations/eu307451> |
| Relacorilant | 21-08-2019 | Relacorilant is a 'glucocorticoid receptor antagonist'. This means that it attaches to the same receptors (targets) that the hormone cortisol attaches to inside cells. When cortisol attaches to these receptors in pancreatic cancer cells it helps them to grow and resist cancer medicines. Relacorilant reduces cortisol’s ability to attach to the receptors, and this is expected to slow the growth of the cancer. | No | <https://www.ema.europa.eu/en/medicines/human/orphan-designations/eu3192191> |
